# Circulating Tumor DNA in Prostate Cancer: A Dual Perspective on Early Detection and Advanced Disease Management

**DOI:** 10.3390/cancers17152589

**Published:** 2025-08-06

**Authors:** Stepan A. Kopytov, Guzel R. Sagitova, Dmitry Y. Guschin, Vera S. Egorova, Andrei V. Zvyagin, Alexey S. Rzhevskiy

**Affiliations:** 1 Scientific Center for Translation Medicine, Sirius University of Science and Technology, 354340 Sirius, Russia; egorova.vs@talantiuspeh.ru (V.S.E.); zvyagin.av@talantiuspeh.ru (A.V.Z.); 2 Institute of Molecular Theranostics, Sechenov University, 119991 Moscow, Russia; sagitova_g_r@staff.sechenov.ru (G.R.S.); rzhevskiy_a_s@staff.sechenov.ru (A.S.R.); 3 Shemyakin-Ovchinnikov Institute of Bioorganic Chemistry, Russian Academy of Sciences, 117997 Moscow, Russia; 4 School of Mathematical and Physical Sciences, Macquarie University, Sydney, NSW 2109, Australia

**Keywords:** prostate cancer, localized prostate cancer, metastatic prostate cancer, liquid biopsy, circulating tumor DNA, ctDNA, biomarkers, precision oncology

## Abstract

**Simple Summary:**

Prostate cancer (PC) diagnosis and monitoring face challenges with the current methods, such as PSA tests and biopsies. Liquid biopsy, specifically analyzing circulating tumor DNA (ctDNA) from bodily fluids, offers a promising non-invasive alternative. This review explores how ctDNA can help manage both early (localized) and advanced (metastatic) PC. For early PC, ctDNA markers can improve detection accuracy and predict recurrence risk. For advanced PC, ctDNA analysis helps track tumor changes, understand treatment resistance, and guide therapeutic choices. While challenges, including the low ctDNA levels at the early stages and test standardization, remain, the technological advances make ctDNA more attractive for clinical applications. Combining ctDNA with medical imaging modalities and other tests paves the way for more precise cancer care, potentially improving patient outcomes.

**Abstract:**

Prostate cancer (PC) remains a leading cause of malignancy in men worldwide, with current diagnostic methods such as prostate-specific antigen (PSA) testing and tissue biopsies facing limitations in specificity, invasiveness, and ability to capture tumor heterogeneity. Liquid biopsy, especially analysis of circulating tumor DNA (ctDNA), has emerged as a transformative tool for non-invasive detection, real-time monitoring, and treatment selection for PC. This review examines the role of ctDNA in both localized and metastatic PCs, focusing on its utility in early detection, risk stratification, therapy selection, and post-treatment monitoring. In localized PC, ctDNA-based biomarkers, including ctDNA fraction, methylation patterns, fragmentation profiles, and mutations, demonstrate promise in improving diagnostic accuracy and predicting disease recurrence. For metastatic PC, ctDNA analysis provides insights into tumor burden, genomic alterations, and resistance mechanisms, enabling immediate assessment of treatment response and guiding therapeutic decisions. Despite challenges such as the low ctDNA abundance in early-stage disease and the need for standardized protocols, advances in sequencing technologies and multimodal approaches enhance the clinical applicability of ctDNA. Integrating ctDNA with imaging and traditional biomarkers offers a pathway to precision oncology, ultimately improving outcomes. This review underscores the potential of ctDNA to redefine PC management while addressing current limitations and future directions for research and clinical implementation.

## 1. Introduction

Prostate cancer (PC) is one of the most common malignant neoplasms in men worldwide [1]. Traditional diagnosis relies on measuring prostate-specific antigen (PSA) levels in the blood, followed by prostate biopsy if cancer is suspected. However, PSA lacks specificity due to elevations in benign conditions (e.g., prostatitis, benign prostatic hyperplasia (BPH)) [2,3,4]. Meanwhile, despite its high specificity [5], biopsy carries risks of complications, such as bleeding, abscess, and even erectile dysfunction [6].

The disease is characterized by significant biological heterogeneity: some tumors are indolent and unlikely to affect life expectancy, while others are aggressive with a high risk of metastasis and mortality. While functional, traditional clinical parameters, such as Gleason score, PSA level, and tumor staging, are often insufficient to predict tumor behavior in early stages. This leads to risks of both overtreatment of indolent disease and undertreatment of aggressive disease. Accurate risk stratification for localized PC (disease confined to the prostate) remains challenging without evidence of spread to other organs or tissues [7].

Therefore, there is an urgent need for additional molecular markers to improve PC risk stratification and enable more personalized treatment approaches. In this context, one of the most promising means is liquid biopsy, a non-invasive technology that allows detection of such markers in various biological fluids, including circulating tumor cells, cell-free DNA, exosomes, proteins, and other molecules secreted by a tumor [8]. Circulating tumor DNA (ctDNA) is of particular diagnostic significance in cancer patients. ctDNA is a fraction of the total cell-free DNA (cfDNA) that is released into the bloodstream or other biological fluids due to cell death [9]. In healthy individuals, cfDNA mainly originates from the apoptosis of normal cells, whereas in cancer patients, a proportion of cfDNA stems from tumor cells. Analysis of ctDNA allows detection of mutations, methylation changes, and other molecular features related to tumor biology. This opens up new possibilities for more accurate diagnosis and personalized therapy while avoiding unnecessary invasive procedures.

## 2. Classification, Conventional Diagnostics, and Staging of Prostate Cancer

Prostate cancer is stratified into two clinically distinct entities: non-metastatic (localized or locally advanced) PC and metastatic PC (mPC). While non-metastatic PC is confined to the prostate gland or adjacent tissues, mPC is defined by disseminated tumor spread to distant sites, including most commonly the bone, lymph nodes, and lungs, and to a lesser extent, the liver, pleura, and adrenals, based on the extensive autopsy study of 1600 PC patients globally [10]. The prevalence of the metastatic sites for mPC and prognostic significance are listed in Table 1. This transition to metastases represents a pivotal shift in the disease biology, prognosis, and therapeutic strategy [11,12]. The lymph nodes and liver metastases often correlate with aggressive disease and poor prognosis compared to the bone-only metastases. The less frequent occurrence of visceral metastases partially relates to the limited access to advanced diagnostics in developing nations, as reviewed in [13]. Even in developed nations, advanced imaging demonstrates persistent underdetection of mPC. In a recent study, Holzgreve et al. demonstrated that conventional imaging methods used clinically are not capturing metastases that are captured by PSMA-PET/CT [14].

The clinical management of prostate cancer is dictated by its disease state, broadly categorized within established frameworks [15,16,17,18,19,20,21,22] as follows:

1. **Localized/locally advanced prostate cancer**: Disease confined to the prostate gland or immediately adjacent tissues (T1-T4, N0/X, M0).
2. **Biochemically recurrent (BCR)/non-metastatic castration-sensitive prostate cancer (nmCSPC)**: Rising PSA following definitive local therapy, without evidence of metastases on conventional imaging (M0).
3. **Metastatic hormone-sensitive prostate cancer (mHSPC)**: Radiographically evident metastatic disease, responsive to initial androgen deprivation therapy (ADT) (M1).
4. **Non-metastatic castration-resistant prostate cancer (nmCRPC):** Rising PSA despite castrate levels of testosterone (<50 ng/dL) and no detectable metastases on conventional imaging (CT/bone scan) (M0).
5. **Metastatic castration-resistant prostate cancer (mCRPC):** Radiographically evident metastatic disease progressing despite castrate levels of testosterone (<50 ng/dL) (M1).

These fundamental states reflect distinct therapeutic contexts and prognoses. Within these broader categories, specific subpopulations with unique biological features or risk profiles exist. For example:

- *High-risk/very high-risk localized* disease represents a subset of **localized/locally advanced prostate cancer**.
- *Oligometastatic* disease can manifest within the **mHSPC** or **mCRPC** states.
- *Aggressive variant prostate cancers (AVPCs)/neuroendocrine prostate cancer (NEPC)* typically arise within the **mCRPC** state.

Understanding both the overarching disease states and the clinically relevant sub-entities within them is crucial for optimizing treatment strategies.

### 2.1. Imaging

Imaging is essential for detecting metastatic spread and guiding prostate cancer management. Key modalities include the following:

1. **Bone scan**: It detects osteoblastic metastases using technetium-99m-labeled diphosphonates. It remains cost-effective and widely available but has low sensitivity for early micrometastases/osteolytic lesions and carries a risk of false positives [23].
2. **CT/MRI**: CT evaluates lymphadenopathy and visceral metastases for TNM staging but lacks sensitivity for subcentimeter lesions [24]. MRI provides superior soft-tissue contrast for spinal/leptomeningeal disease and local recurrence but is less sensitive than PSMA PET/CT for small metastases [24,25].
3. **PSMA PET/CT**: It targets prostate-specific membrane antigen (PSMA) with Ga-68/F-18 tracers. It detects micrometastases (<10 mm), altering management in ~30% of cases by identifying oligometastatic disease or upstaging. It is recommended for high-risk staging and biochemical recurrence despite limited availability and higher cost [26,27,28].
4. **FDG PET/CT**: It measures glucose metabolism but has limited utility in prostate adenocarcinoma due to low FDG avidity. It is primarily reserved for aggressive neuroendocrine subtypes [29].
5. **PSMA PET/MRI**: It combines PSMA PET with MRI’s soft-tissue resolution, improving pelvic/spinal lesion detection. However, it suffers from limited availability, longer scan times (60–90 min), and higher costs [25].
6. **Whole-body MRI**: It provides radiation-free assessment of bone/visceral metastases, ideal for young patients or repeated monitoring. It is effective for bone-dominant disease but has longer scan times (30–60 min) and inferior sensitivity for small lymph node metastases vs. PSMA PET/CT [30].

While conventional imaging (bone scan, CT, MRI) is foundational, PSMA PET/CT offers superior sensitivity for osseous/soft-tissue metastases and frequently alters clinical management [26,27,28]. PSMA PET/MRI enhances soft-tissue detail but has accessibility challenges. FDG PET/CT is niche (neuroendocrine PC), and whole-body MRI provides a radiation-free alternative. Modality selection balances performance, availability, cost, and patient factors (Table 2) [31].

### 2.2. Biomarkers

While imaging (e.g., PSMA-PET, CT, MRI) provides anatomical and functional insights into PC, it faces limitations in sensitivity for microscopic disease, radiation exposure, and cost [32]. This underscores the need for complementary strategies. Liquid biopsy, particularly ctDNA analysis, offers a minimally invasive window into tumor biology, enabling real-time monitoring of disease burden, heterogeneity, and treatment response [8,33]. Traditional serum biomarkers like PSA, though integral for screening and monitoring, suffer from low specificity (20–40%) due to elevation in benign conditions (e.g., BPH, prostatitis) [2,3,4,34]. PSA also fails to detect ~25% of mCRPC progression (e.g., neuroendocrine differentiation) and correlates poorly with metastatic burden in bone-dominant disease [35,36,37]. Alkaline phosphatase (ALP), reflecting bone turnover, provides prognostic value in mCRPC/HSPC but lacks specificity due to elevation in liver disease or fractures [38,39,40]. Hemoglobin (Hb) aids prognostic models but is nonspecific for cancer progression [41,42,43,44]. Emerging protein biomarkers (e.g., OPN, OPG, and BSP for bone metastasis; AR-V7 for ARSI resistance; and CgA/NSE for neuroendocrine differentiation) show promise but require further validation [45,46,47,48,49,50,51,52,53,54,55,56,57,58,59]. Circulating tumor cells (CTCs) offer prognostic value in mCRPC (e.g., ≥5 CTCs/7.5 mL = poor OS) and can guide therapy (e.g., AR-V7+ predicts taxane benefit over ARSI) but have low detection rates in localized disease and platform-dependent heterogeneity [60,61,62,63,64]. These limitations highlight the potential for ctDNA to overcome specificity gaps and provide dynamic molecular insights.

### 2.3. Biopsy in Prostate Cancer

Prostate biopsy remains the gold standard for histopathological diagnosis, Gleason grading, and molecular characterization (e.g., HRR defects for PARPi eligibility) [65,66]. However, its invasiveness carries risks of sepsis (TR approach: 1–4%) and other complications [6,67]. Sampling bias due to tumor heterogeneity/multifocality may yield false negatives or underestimate aggressiveness, particularly for anterior tumors [65,68]. Repeat biopsies for active surveillance increase patient burden [67]. In mPC, bone metastases (the most common site) are difficult to biopsy and often yield degraded/inadequate tissue for genomic profiling [69,70]. Single biopsies also fail to capture spatial heterogeneity or clonal evolution [58]. These challenges underscore the need for less-invasive alternatives like ctDNA analysis to address spatial–temporal heterogeneity and complement tissue sampling.

In summary, prostate biopsy is a cornerstone of PC management, enabling histopathological diagnosis, risk stratification, and molecular characterization. However, its inherent limitations highlight the growing importance of integrating novel technologies to refine precision oncology approaches in both localized and advanced diseases [71,72].

**Figure 1:**
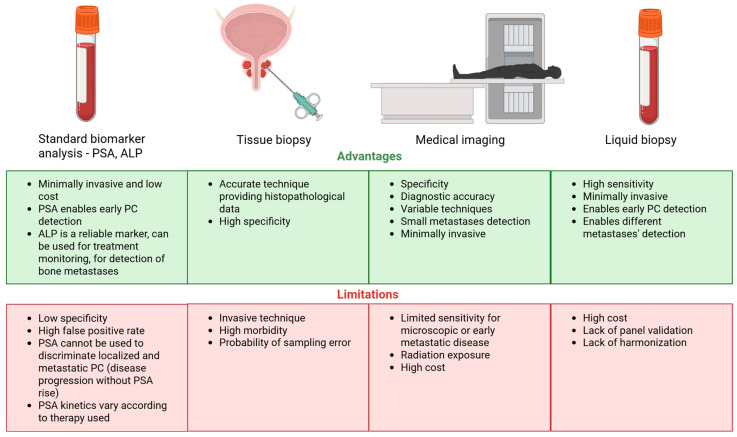
Summary on the diagnostic approaches in PC.

A graphical summary of current diagnostic strategies is provided in Figure 1.

## 3. ctDNA in Localized Prostate Cancer

### 3.1. Early Detection

The analysis of circulating tumor DNA is emerging as a valuable tool in the diagnosis of prostate cancer. It allows tumor-specific mutations, aberrant methylation patterns, and cell-free DNA fragmentation profiles to be detected, as well as providing insights into transcriptomic and broader epigenetic alterations. Even in locally advanced prostate cancer, where ctDNA levels are usually low, the presence of ctDNA may be highly informative prognostically [73].

In the study by Chen et al. [74], using the cfMeDIP-seq method, the cfDNA methylome discriminated localized from metastatic PC with 98.9% accuracy. Even at low tumor DNA fractions (<2%), cfMeDIP detected characteristic epigenetic signatures, including hypermethylation of the NR3C1 promoter, which is associated with immune suppression and poor prognosis. Brikun et al. [75,76] demonstrated the efficacy of methylation panels for PC diagnosis: in one study, non-invasive urine testing achieved 94% sensitivity and 71–76% specificity. The authors confirmed the utility of markers such as GSTP1 and APC in differentiating malignant from benign tissue. While GSTP1 and RASSF1 are traditionally considered epigenetic PC markers, Aykanli et al. (2024) [77] found RASSF2 to be the most diagnostically significant (69% sensitivity). Combining all three markers increased specificity to 83% but decreased sensitivity to 8%.

An alternative approach to the molecular diagnosis of PC is the assessment of the cell-free DNA integrity index (cfDI). This parameter reflects the ratio of long to short cfDNA fragments. The main principle behind the measurement of cfDI is based on the fact that normal cells primarily undergo apoptosis, releasing short DNA fragments of approximately 200 bp in length, whereas tumor cells often die by necrosis or autophagy, generating a wider range of fragments, including longer fragments than typically seen in apoptosis [78]. Casadio et al. (2013) [79] showed that cfDI levels in the urine of PC patients were significantly higher than in healthy volunteers, with a diagnostic accuracy of AUC = 0.80 (sensitivity: 0.79; specificity: 0.84). Feng et al. (2013) [80] also confirmed the applicability of cfDI in plasma cfDNA analysis. The authors showed that the ALU 247/115 ratio was significantly higher in PC patients than in patients with BPH. This significant difference remained even in patients with PSA levels above 4 ng/mL (sensitivity: 81.7%; specificity: 78.8%). Further supporting the diagnostic and prognostic relevance of cfDI, Arko-Boham et al. (2019) [81] reported elevated cfDI values in PC patients compared to healthy individuals (1.00 vs. 0.67; *p* = 0.02), with progressive increases correlating with tumor stage: 0.83 in stage I, 1.50 in stage II, and 2.00 in stage III. However, a more recent study by Condappa et al. (2020) [82] found no statistically significant differences in cfDNA concentration or integrity between PC and BPH patients (cfDI: 0.62 vs. 0.67; *p* = 0.342), nor any meaningful associations with clinical parameters such as PSA levels or Gleason score. These findings may be limited by the small cohort size (11 PC patients and 9 BPH patients), emphasizing the need for larger, standardized studies. Thus, cfDI is a promising non-invasive biomarker that can be used for both initial diagnosis and risk stratification. The advantages of the method include its simplicity, reproducibility, and relative affordability. However, its clinical application requires careful standardization of both primer panels and amplification protocols, as well as the entire analytical process, including sample processing, DNA isolation, and data interpretation procedures.

Temilola et al. (2023) [83] performed whole-exome sequencing of urinary cfDNA in men of African descent. They identified mutations in the BRCA1, ERCC6, ARHGAP21, and ADAMTSL3 genes that reliably distinguished PC from BPH patients. The authors noted that such genetic alterations were not included in standard PC diagnostic panels but had high diagnostic significance in this population. These findings highlight the importance of developing ethnically specific biomarkers to improve diagnostic accuracy in under-represented patient groups.

Another promising direction in PC diagnostics is the analysis of cfDNA in seminal plasma, as it contains molecules directly secreted by the prostate and may serve as a convenient and informative source of biomarkers. Moreover, seminal fluid contains significantly higher cfDNA concentration than blood (Ponti et al., 2018) [84]. Ponti et al. [84,85,86] performed fluorometric and electrophoretic analyses of cfDNA isolated from semen of PC patients and healthy men. They found that cfDNA levels were significantly higher in PC patients. They also reported distinct differences in DNA fragmentation patterns: PC patients had longer fragments and greater length heterogeneity than BPH patients and healthy donors. Several studies have focused on epigenetic analysis of cfDNA in seminal fluid. For example, Zhang et al. (2015) [87] found that promoter methylation of the RARβ2 gene was significantly higher in ejaculate samples from PC patients compared to BPH patients. Meanwhile, Skara et al. (2023) [88] showed that methylation of the CpG1 region of the CAV1 gene in seminal plasma cfDNA was higher in BPH patients than in PC patients, with a diagnostic accuracy (AUC 0.63) exceeding that of PSA (AUC 0.52). Furthermore, CpG1 methylation allowed the differentiation of BPH and indolent tumors (Gleason 1) from potentially more aggressive PC forms (AUC 0.72). Abramovic et al. (2024) [89] showed that methylation levels of the LGALS3 gene in seminal plasma cfDNA were significantly higher in PC patients than in those with BPH (sensitivity 56.4%, specificity 70.4%). Notably, LGALS3 methylation levels in blood were not statistically different between the groups, and PSA did not effectively discriminate between tumors of diverse aggressiveness.

Thus, the evidence suggests that even in localized forms of PC, ctDNA can be detected and used for non-invasive diagnosis, risk assessment, and personalized stratification. Combined approaches that integrate methylation status, mutations, fragmentation profiles, and multimodal circulating cfDNA analysis from different biological fluids show the most significant potential.

A summary of the diagnostic performance of key cfDNA biomarkers in localized prostate cancer is presented in Table 3.

### 3.2. Risk Stratification

After initial diagnosis, accurate risk stratification is critical for selecting appropriate treatment strategies that balance active surveillance with aggressive intervention. In current clinical practice, stratification is based on three primary parameters: PSA levels, Gleason score (from biopsy results), and clinical tumor staging (cT, cN, cM) [90]. These data are used in models such as the D’Amico classification and the NCCN risk groups, which classify patients into low-, intermediate-, and high-risk groups. However, even with identical PSA levels and Gleason scores, tumors can behave differently: some remain indolent while others progress rapidly. Consequently, increasing attention is being paid to molecular stratification, including the analysis of ctDNA and other biomarkers.

In the study by Fei et al. (2023) [91], preoperative ctDNA status in patients with non-metastatic PC was found to be an independent prognostic marker for BCR. Patients who were ctDNA-positive had a median recurrence-free survival of 8.2 months, whereas ctDNA-negative patients exhibited minimal recurrence (HR = 0.14, *p* < 0.01). These differences persisted even in subgroups of patients with early-stage disease (T1–2 and N0), highlighting the importance of ctDNA in preoperative risk stratification. The study by Pope et al. (2024) [92], using the highly sensitive INVAR method, also confirmed the prognostic role of ctDNA. In 16% of patients with localized PC, ctDNA was detected preoperatively and was associated with a higher likelihood of both BCR (HR 3.3; *p* = 0.0001) and metastasis (HR 2.8; *p* = 0.0055). In addition, in multivariate analysis, ctDNA was a stronger predictor of recurrence than PSA level and pathological stage.

Mutational analysis of ctDNA may also be considered for risk stratification. In the study by Stitz et al. (2024) [93], AR alterations were identified in 26.1% of patients with CRPC but only in 3.7% of patients with HSPC. These findings support the potential use of AR profiling as an early predictor of developing a hormone-resistant phenotype, even at the localized stage of the disease.

Taken together, these data confirm that liquid biopsy is not only a diagnostic tool but also a powerful method for molecular risk stratification in localized PC, with potential for integration into preoperative planning and personalized therapy.

### 3.3. Post-Treatment Monitoring

Circulating tumor DNA is being actively investigated as a tool for monitoring residual disease and predicting recurrence after radical treatment for localized PC. In the aforementioned study by Zhang et al. (2025) [94], ctDNA-positive status after radical prostatectomy following neoadjuvant therapy with darolutamide and androgen deprivation was a significant prognostic marker: 80% of ctDNA-positive patients experienced disease progression, whereas only 5% of ctDNA-negative patients did. The predictive sensitivity was 80% and specificity was 95%, highlighting the potential of ctDNA as a marker of minimal residual disease (MRD) and a basis for personalized monitoring.

An innovative approach to cfDNA monitoring was proposed by Alves et al. (2025) [95] using the nonlinear optical Z-scan technique. The authors used the optical parameter θ/cfDNA, which reflects changes in the refraction of laser light depending on the concentration and structure of cfDNA in the sample. The study included patients following chemotherapy, some of whom later experienced BCR. It was shown that θ/cfDNA values were statistically significantly higher in patients with recurrence than in those without signs of progression (mean values: 0.210 vs. 0.152, *p* = 0.036), indicating the potential of the method to detect residual or recurrent disease at an early stage.

In the study by Weiss et al. [96], whole-genome sequencing (WGS) of cfDNA was performed in patients with aggressive PC. The study included patients with both localized and advanced PC who had undergone radical treatment, including prostatectomy. Patients with poorer clinical outcomes were more likely to have mutations in BRCA2, ATM, and CDK12, as well as high tumor mutation burden (TMB) and microsatellite instability (MSI). Notably, some of these alterations were detected in cfDNA but not in biopsy analyses, highlighting the ability of cfDNA to capture molecular heterogeneity and tumor evolution after therapy.

Thus, ctDNA is a promising non-invasive tool for post-radical treatment monitoring, allowing detection of MRD, prediction of recurrence, and early adjustment of surveillance and treatment strategies.

### 3.4. Technical Considerations

Developing reliable liquid biopsy methods for localized PC faces several technical challenges. As shown by Hennigan et al. [97], ctDNA often remains undetectable in patients with localized PC, even when advanced sequencing techniques are utilized. This highlights the need to further improve the sensitivity of existing technologies.

In the review by Gorgannezhad et al. (2018) [98], it was noted that the most promising approaches include digital PCR and next-generation sequencing (NGS), which show higher sensitivity with minimal concentrations of tumor material. Recent studies support this. For example, the work of Stitz et al. [93] demonstrates the capabilities of multiplex ddPCR analysis for the simultaneous detection of transcripts (AR-V7, KLK3), point mutations, and amplifications of the AR gene. The method demonstrated high sensitivity (limit of detection ~3–8 copies/mL), specificity, and reproducibility, as well as the ability to use cell-free RNA, extending the potential of liquid biopsy analysis beyond cfDNA.

The cfMeDIP-seq method (methylated DNA immunoprecipitation followed by sequencing) demonstrated high sensitivity in the study by Chen et al. [74]. It discriminated between localized and metastatic forms with up to 98.9% accuracy, even with ctDNA fractions as low as 2%.

Assessing the fragment composition of cfDNA is also promising: studies by Casadio, Feng, and others have shown that the ratio of long to short fragments (cfDI) can serve as an additional biomarker [79,80]. Such parameters require high-precision fragment size analysis and standardized calculation algorithms.

Overall, the technical implementation of liquid biopsy in clinical practice requires consideration of method sensitivity, panel validation, and alignment with other diagnostic tools (e.g., imaging, tissue biopsy).

### 3.5. Challenges and Future Directions

Despite significant advances in liquid biopsy, its application in localized PC faces several challenges. Key limitations and corresponding mitigation strategies are summarized in Table 4. A primary obstacle is the low tumor burden in localized disease. Circulating ctDNA levels often fall below the sensitivity thresholds of most platforms in localized forms [98]. Studies by Kluge et al. [99,100] have shown that ctDNA is virtually undetectable in localized PC and does not correlate with tumor volume. The authors emphasize the need to combine liquid biopsy with imaging modalities such as [^68^Ga]Ga-PSMA−11 PET/CT, especially in the context of surveillance and risk stratification.

Molecular heterogeneity and phenotypic plasticity of tumors require multi-analytical approaches. For example, cfDNA reflects mutational profiles but does not provide direct information on the transcriptional activity of the tumor. The study by Ding et al. [101] showed that EV-DNA and EV-RNA (extracellular vesicular components) may provide more accurate insights into the current biological state of the tumor, including resistance to therapy.

Limited tissue availability in some patients, particularly those on active surveillance, makes liquid biopsy particularly valuable. The study by Weiss et al. [96] showed that WGS of cfDNA can identify driver mutations, mutational signatures, MSI, and tumor mutational burden in aggressive forms of PC, which could be adapted to earlier stages with appropriate platform sensitivity.

Standardization of pre-analytical procedures also remains crucial. The work of Bonstingl et al. [102] demonstrated that strict adherence to international ISO and CEN standards ensures the comparability of results between laboratories. Factors such as hemolysis, time to centrifugation, storage methods, and transport can all influence results, and standardized procedures help to minimize their impact.

**Table 4:** Key Technical Challenges and Solutions in Liquid Biopsy Based on Cell-Free DNA for Localized Prostate Cancer.

Challenge	Description	Potential Solutions/Technologies	References
**Low ctDNA concentration**	ctDNA levels often fall below detection thresholds in early-stage PC	Highly sensitive methods: ddPCR, NGS, cfMeDIP-seq	Hennigan et al., Chen et al. [74,97]
**Limited sensitivity of standard assays**	Conventional PCR/sequencing may miss low-frequency variants	Use of multiplex ddPCR; optimized amplicon panels; integration of cfRNA and other biomarkers	Stitz et al. [93]
**Pre-analytical variability**	Sample degradation due to handling/storage/processing errors	Compliance with ISO 20186–3, CEN/TS 17390–3 standards	Bonstingl et al. [102]
**Lack of protocol standardization**	Inconsistent results across labs; difficult to compare studies	Harmonized workflows, SOPs, multicenter validation	Gorgannezhad et al. [98]
**Limited scope of cfDNA**	cfDNA reflects mutations but not dynamic gene expression	Combine with cfRNA, EV-DNA/RNA, or exosomal analysis	Ding et al. [101]

However, modern technologies are enabling novel approaches. For example, the study by Liu et al. (2024) [103] introduced the DirectHRD method, which can classify tumors with homologous recombination deficiency using cfDNA at a tumor fraction as low as 1%. Similarly, Alves et al. [95] demonstrated the sensitivity of the optical Z-scan technique to differences in cfDNA during recurrence, which could become an accessible tool for dynamic monitoring in resource-limited settings.

Finally, the development of population-specific biomarkers is becoming increasingly important. Temilola et al. [83] showed that the urinary cfDNA mutation profile of African PC patients differs from standard panels, highlighting the need for individualized approaches to screening and surveillance.

Prospectively, large-scale prospective clinical trials are needed to validate the use of liquid biopsies for localized prostate cancer, particularly when compared with or combined with modern imaging and histological methods. Integrating liquid biopsy into multimodal diagnostic algorithms that combine molecular, imaging, and clinical parameters will be essential for reliable personalized treatment. The most promising strategies will ultimately include composite biomarker panels that combine mutation profiling, methylation status, fragmentation patterns, and transcriptomic signals. These panels will be designed to accommodate feasibility and real-world variability.

## 4. ctDNA in Metastatic Prostate Cancer

The behavior of metastatic PC differs significantly from localized forms. While the localized PC is often characterized by relatively high survival rates following standard treatment regimens, mPC treatment outcomes are daunting, with a 5-year survival rate of approximately 30% owing to its aggressive nature and high propensity for metastasis [104]. mPC is known to be refractory to many drugs, the benefits of which for prolonged survival remain ambiguous. The mPC resistance patterns are divided into AR-driven and non-AR-driven. Most castration-resistant tumors are AR-driven, while non-AR-driven cases are less common and less characterized. A significant form of the non-AR-driven disease is the neuroendocrine (CRPC-NE) phenotype observed in 15–20% of the advanced cases and associated with the loss of TP53 and RB1 genes [105].

Tumors from the high-volume diseases have the higher fraction of the altered genomes. The genomic instability correlates with oncogenic changes in the genes of the repair system, particularly HRR (BRCA1/2, among others), as well as in NOTCH and cell cycle genes. Mutations in TP53, MYC, SPOP, and WNT genes are common. AR alterations are most strongly associated with the development of the castration-resistant phenotype. Common genetic changes observed in mCRPC involve such genes as AR, TP53, PTEN, RB1, BRCA1–2, and CDK12. These alterations impact essential signaling pathways, cell cycle regulation, and DNA repair [106]. In CRPC-NE, compared with CRPC adenocarcinoma, cancer-specific mutations or copy-number changes are only modestly enriched, but DNA methylation is more extensive, as shown using ctDNA analysis [105,107].

Progression of PC from localized to metastatic disease is followed by significant changes in ctDNA levels, ranging from 1% at the diagnosis stage or in patients with localized disease to 90% in men with high-volume progressive metastases in mCRPC [106]. In localized disease, ctDNA represents 0.1–10% of total circulating cfDNA, with plasma levels ranging from 10 to 100 ng/mL [108], whereas in the case of advanced disease, it reaches 80–90% [109]. The limit of detection (LoD) of NGS, measured as approximately 10 ng/mL, is applicable to the metastatic PC, but not to the localized PC due to lower ctDNA levels. As the disease progresses to the metastatic stages, the ctDNA fraction (ctDNAF) increases accordingly, indicating the higher tumor burden and more aggressive tumor behavior [106]. At the same time, therapy can cause a reduction of the biomarker levels, including the ctDNAF [110].

The most common methods for analyzing ctDNA in liquid biopsy materials include NGS and ddPCR. These methods allow identification of the point mutations, copy number changes, and structural rearrangements, which have been used for detecting recurrent genetic alterations in genes such as AR, TP53, BRCA1–2, MYC, PTEN, and many others. Some of these genes are important for therapy selection because they are associated with the expression of the drug targets. For example, poly(adenosine diphosphate-ribose) polymerase (PARP) inhibitors are used for the treatment of patients with DNA damage repair (DDR) defects. Immune checkpoint inhibitors have been proposed as a potential treatment option for patients with mismatch repair deficiency (dMMR) [111].

### 4.1. Liquid Biopsy in Advanced Disease

The conventional approach to diagnosing and monitoring cancer primarily relies on tissue biopsies, which have several drawbacks [69,70,112]. Liquid biopsy, including cfDNA analysis, has emerged as an alternative to tissue biopsy for assessing the molecular subtypes of tumors. ctDNA levels in blood correlates with the tumor load, and the concordance between ctDNA genomic characteristics with the tumor increases as the disease progresses, from approximately 50% in localized PC to 80–90% in metastatic PC [109]. Table 5 summarizes the differences in the ctDNA characteristics between the localized and metastatic prostate cancers.

### 4.2. Clinical Applications

#### 4.2.1. Prognostication

Recent studies have highlighted an important role of the liquid biopsy for identifying prognostic and predictive biomarkers for PC. The ctDNAF—the percentage of cfDNA released into the bloodstream by the tumor—has been proposed as a potential consensus marker for mCRPC prognosis. This metric is incorporated into commercial assays, but its utility is still investigated. Among patients with mCRPC treated with ARSI or taxanes, the undetectable levels of ctDNAF at the baseline (below LoD for targeted NGS) predicted significantly better PFS and OS, suggesting a basis for treatment and monitoring de-escalation [116]. Low-pass WGS of ctDNA in mCRPC patients undergoing ARSI treatment demonstrated that the high ctDNAF and copy number alteration burden at the baseline were associated with the shorter PFS and OS, as well as the poor ARSI treatment response, particularly in the cases of chr3q amplification, chr13q deletion, chr18q deletion, and chrXq amplification [117]. The results from two clinical trials have been analyzed to assess the potential of using ctDNAF (measured via targeted NGS) as an independent biomarker. The analysis revealed a strong correlation with the known prognostic and therapy response indicators such as OS, PFS, PFS-PSA, and PSA. Notably, the same study reported the development of a machine learning tool that could predict whether the ctDNA analysis would be superior in comparison with the tissue biopsy analysis based on the results of several laboratory and radiographic tests [118]. A similar correlation between a high baseline ctDNAF and worse OS, radiographic PFS (rPFS), and PFS-PSA has been found using the low-pass WGS [119].

It has been demonstrated that ctDNAF levels in mHSPC were generally significantly lower, where the characteristics of ctDNA in mHSPC and mCRPC were compared. This finding suggests that ctDNAF cannot be used as an independent parameter, but can supplement the other metrics to improve predictions [120]. In a study combining liquid biopsy and functional imaging parameters, a significant association between ctDNAF in blood plasma and metabolic tumor volume have been demonstrated in mCRPC patients, as well as a correlation between both parameters and tumor metabolic activity. Both parameters appeared predictive of OS and PFS, leading to a hypothesis that their combined use could improve the accuracy when predicting a response to therapy [121]. A comparison of ctDNAF and PSMA-targeted PET results in the locally recurrent and metastatic PSMA-positive HSPC and CRPC with very low PSA levels has shown that both assessments were independently prognostic for survival outcomes. However, the PSMA PET imaging outperformed the ctDNA analysis in detection, likely due to the low ctDNAF levels at the minimal tumor burden. This again highlighted the dependence of ctDNAF on tumor metabolic activity and, accordingly, the number of metastases, which was observed to be higher in mCRPC [122].

A limited number of studies have addressed gene-specific correlations via ctDNA. Most results have indicated some influence of known genetic variations in the genes important for PC, particularly when targeted sequencing or ddPCR was employed. Occasionally, the whole-genome or whole-exome sequencing has identified new variations, but these have typically been in the genes already recognized as significant for PC. BRCA mutations have often been associated with poor prognostic features and clinical outcomes. Combined analysis of tumor tissue and ctDNA has shown that patients with any pathogenic variations in BRCA1, BRCA2, CDK12, TP53, PTEN, or RB1 experienced the shorter time to CRPC and that the accumulation of these variations increased a risk of developing CRPC. In addition, the genomic alterations in AR, TP53, RB1, and PTEN have been associated with poor clinical outcomes [109]. CtDNA analysis has also revealed associations between several genomic alterations and clinical outcomes, such as PFS in ARSI and OS in mCRPC patients [123]. A substantial number of individual variations were relevant factors in therapy selection and are discussed in the following subsection.

Simultaneous determination of genomic and epigenomic (methylation patterns) characteristics in the most advanced variants of the disease has enabled the discovery of several significant differences essential for prognostic purposes. Beltran et al. [105] demonstrated that alterations involving RB1, TP53, and CYLD were more common in the CRPC-NE patients, while AR alterations were more prevalent in the CRPC-adenocarcinoma patients. In contrast, DNA repair gene aberrations involving BRCA1, BRCA2, and ATM showed no significant differences in frequency. Many of the identified alterations were associated with better or worse prognoses, with certain genomic alterations, such as AR, TP53, and RB1 exhibiting different prognostic values depending on the histologic subtype. Combination of the genomic and epigenomic alterations applied to ctDNA can be capable of identifying the more aggressive conditions, such as CRPC-NE. Franceschini et al. [124] have found a relationship between the ctDNA content and clinical outcomes for patients with aggressive variants of CRPC and NEPC. The authors have developed a targeted ctDNA methylation assay for CRPC-NE detection, which allowed the quantitation of the tumor volume and the determination of the phenotype. The methylated fraction of ctDNA was associated with the clinical outcomes for the patients with CRPC and CRPC-NE, facilitating the patient stratification. High fractions of the AR binding sequences in ctDNA and hypomethylation of the corresponding segments were also associated with the more aggressive cases of mCRPC, as demonstrated by the simultaneous genomic and methylomic ctDNA analysis [113].

A recently developed cfMeDIP-seq method has enabled efficient analysis of ctDNA methylomes. Chen et al. have identified several significant methylation patterns in the ctDNA from patients with localized and metastatic prostate cancers. Their analysis has allowed the differentiation of the disease subtypes with an approximately 99% prediction accuracy, and the results showed strong correlations with the clinical outcomes. Additionally, this method offered a cost advantage over the traditional bisulfite sequencing [74].

Overall, the most common ctDNA-based indicator was the total ctDNAF. The fractions of individual genomic and epigenomic variations were less frequently used as the key or secondary objects of analysis in many studies, often clinical, involving samples ranging from several tens to thousands of patients [113,125,126,127,128]. The potential usefulness of these indicators was manifested by the emergence of several commercial tests. However, many authors have noted a need for further research. The recent American Society of Clinical Oncology (ASCO) recommendations suggested the use of ctDNA as an alternative to metastatic tissue biopsy, primarily when the metastatic lesions were unavailable or in case of their ambiguous analysis [115].

#### 4.2.2. Therapy Selection

Poly(adenosine diphosphate-ribose) polymerases (PARPs) are enzymes that catalyze poly-ADP-ribosylation, a post-translational modification of proteins. Members of the PARP−1 and PARP−2 superfamily are involved in the HRR of DNA damage. Although the BRCA proteins (BRCA1 and BRCA2) are unrelated to each other, both play crucial roles in the homologous repair of double-strand breaks as well [129]. Many common BRCA mutations lead to genetic instability due to errors in repair by the other pathways, significantly increasing a risk of developing various cancers, including PC [130]. The dysfunction of both PARP and BRCA results in a phenomenon known as “synthetic lethality,” where the cell dies due to the simultaneous dysfunction of two or more genes, while the dysfunction of either gene alone does not cause the cell’s death. The BRCA mutations lead to repair errors, and the PARP inhibition allows replication of these errors, resulting in the accumulation of the DNA damage that ultimately kills the cell. This mechanism has formed the basis for the PARP inhibitor therapy for treatment of cancers associated with the BRCA mutations [131]. Additionally, the mutations in the other genes related to homologous recombination, such as ATM, PALB2, and RAD51, may also warrant further investigation [132].

In cases of the homologous recombination deficiency associated with the mutations in these genes, tumors become more sensitive to treatment with PARP inhibitors (PARPis) such as olaparib, rucaparib, niraparib, and talazoparib [133,134]. The main indication for prescribing these drugs is detection of the relevant mutations, including those identified via ctDNA analysis. There is a category of the diagnostic tests called companion diagnostics (CDx) that identifies the tumor susceptibility to a specific drug. The FoundationOne Liquid CDx test detects mutations in the BRCA1, BRCA2, and ATM genes in ctDNA, enabling informed treatment decisions favoring olaparib or rucaparib [135]. BRCA mutations can also be detected by the Guardant360 CDx and Illumina TruSight Oncology 500 tests, although these tests are not claimed to be intended for use in prostate cancer [136,137]. Note that the PARPi treatment often leads to the development of resistance due to secondary mutations [132].

Sequencing of ctDNA allows detection of the drug-sensitizing and drug-resistant mutations and is utilized for identifying MRD after surgery and early relapse based on the mutational or methylated DNA abnormalities [138]. Detectable AR variations in ctDNA from the blood serum have been proposed as potential factors in treatment decisions between ARSI and taxanes [139]. A prospective phase 2 study, involving a cohort of patients with metastatic mCRPC, demonstrated that the AR amplification induced a resistance to the radioligand therapy with Lutetium−177-PSMA [140]. A recent study has evaluated a modified ctDNA sequencing-based rapid genome-wide aneuploidy screening (mFAST-SeqS) system in the patients with mCRPC. This study concluded that a high blood aneuploidy burden at the baseline was associated with the poor response to ARSI but not taxanes [141].

Despite the availability of the certified commercial tests, their clinical utility is still under evaluation by healthcare professionals. The recent ASCO guidelines highlight several issues, including the search for variants of uncertain significance, which may be reclassified as benign or pathogenic in the future and may have clinical and/or familial implications. Additionally, many genes identified through NGS are of uncertain significance or remain candidates solely as the prognostic biomarkers [115].

#### 4.2.3. Post-Treatment Monitoring

The most common parameter for dynamic monitoring of the PS treatment response is PSA, although it is not entirely reliable. An attractive alternative to PSA is the epigenetic modification of ctDNA, particularly methylation [142]. Studies assessing the prognostic potential of ctDNA methylation markers have not demonstrated applicability for differentiating conditions other than the metastatic variants with a large tumor volume, as other conditions are associated with insufficient ctDNA concentration [127,143]. A retrospective study comparing multiple ctDNA methylation markers with PSA levels and PSMA PET/CT results in patients with various localized and advanced cancer subtypes undergoing different therapies has indicated the promise of ctDNA methylation markers for minimally invasive detection and prognosis of CRPC. These markers can serve as a potential alternative for monitoring response dynamics in patients with any PC types, especially when PSA levels are low. PSA is generally more effective in this role except in cases of advanced metastatic CRPC [99].

Among patients with mCRPC treated with abiraterone, an increase in ctDNAF in the first sample after the start of therapy was associated with the rise in PSA and with the elevated risk of early radiographic progression. Therefore, ctDNAF analysis is practical as a part of routine monitoring of the treatment response to predict outcomes [144]. In patients with mCRPC initially treated with ARSI, early changes in ctDNAF were linked to the development of resistance and poor survival, suggesting that such changes can warrant early therapy modification or intensification [145].

The ASCO clinical guidelines highlight the utility of monitoring ctDNAF for dynamically tracking emerging drug resistance during treatment and adjustments related to intensification, deintensification, and other treatment adaptations. However, the individual biomarkers require further validation in clinical trials [115].

### 4.3. Technical Advances

The most common technology for detecting sequences in ctDNA is sequencing, which includes both Sanger and NGS. The prevalent sequencing techniques are WGS, exome or transcriptome sequencing [105], targeted sequencing [118,124], and low-pass sequencing (low-pass WGS) [117]. Epigenomic data obtained through bisulfite sequencing and immunoprecipitation are valuable as well. A combination of the genomic and epigenomic information yields extensive data useful for identifying biomarkers [113]. The ddPCR method has also been utilized [121]. Specific examples of the technology applications are presented in Section 4.2. of this review: Clinical Applications.

One of the innovative approaches in ctDNA sequencing is methylation analysis. The NEMO panel based on bisulfite sequencing enables simultaneous assessment of the tumor burden and determination of the neuroendocrine phenotype while using a minimal number of informative regions [124]. A similar outcome has been reported using the immunoprecipitation method [107]. Xenotransplantation of patient-derived xenografts into mice has helped address the challenge of low ctDNA content in blood samples during concurrent ctDNA, ctRNA, and epigenome sequencing. This approach has enabled study of the transcriptional regulation in CRPC and contributed to the establishment of a model for identifying phenotypes, such as NEPC, using ctDNA-based analysis [146].

New sequencing methods have demonstrated promising results in the analysis of ctDNA. A recently developed cfMeDIP-seq method has enabled highly efficient analysis of the ctDNA methylomes, allowing for the distinction of different disease phenotypes with 99% prediction accuracy. Since cfMeDIP-seq does not require chemical treatment of DNA, it tolerates the lower cfDNA input and surpasses the bisulfite sequencing in cost-effectiveness [147]. Comparative data exist between cfMeDIP-seq and the presumably more robust cfMBD-seq (cell-free methyl CpG-binding domain protein sequencing) for detection of the metastatic and localized cancers. This comparison has been conducted by the authors in conjunction with a machine learning model they developed, which identified methylation patterns in the cfDNA capable of distinguishing cancerous from non-cancerous subjects [148].

Nanopore sequencing has accelerated the analysis of somatic gene structural variations compared to whole-genome sequencing, the relatively low production rate of which largely precluded tracking tumor burden prior to the initiation of patient treatment. Also, this technology has the potential to surpass WGS in cost-effectiveness, with the authors estimating a five-fold improvement [149]. The combination of nanopore sequencing with rolling circle amplification (RCA), termed Nanopore Rolling Circle Amplification-enhanced Consensus Sequencing (NanoRCS), has enabled a highly sensitive detection of ctDNA fractions, achieving a limit of detection (LoD) of 0.24%. This sensitivity exceeds that of the conventional shallow sequencing methods, which reported the LoD values in the range of 2.5–10% ctDNA fraction [150,151] due to the more reliable single nucleotide polymorphism (SNP) detection [152]. Additionally, the efficacy of nanopore sequencing has been demonstrated for the rapid (<24 h) analysis of copy number aberrations and cfDNA fragmentation patterns.

A popular emerging application of sequencing is the analysis of ctDNA fragmentation patterns, which provides valuable information on the methylome and transcriptome and enables, for example, effective differentiation of the phenotypes [146]. However, fragmentation analysis typically requires costly whole-genome sequencing (WGS), which conflicts with the current FDA requirements. To circumvent this limitation, the authors have proposed a machine-learning algorithm for analyzing the fragmentation patterns within the first coding exon of the standard targeted cfDNA cancer gene panels. This approach was evaluated in two independent cohorts of oncology patients with various cancer types, including prostate cancer. The model demonstrated notable merits, including high sensitivity (LoD for ctDNA fraction ~0.1%), the ability to precisely distinguish tumors from normal samples even at the low ctDNA fractions, and the capability to differentiate certain advanced disease variants, such as adenocarcinoma and NEPC [114].

Overall, the application of new sequencing technologies represents a promising and forward-looking avenue for further research. This includes the adoption of the methods previously applied to other cancer types, save prostate cancer, such as enzymatic methyl sequencing (EM-seq) [153], as well as the modification and combination of existing approaches—for example, the simultaneous detection of DNA methylation and genetic variations [154].

CDx tests are notable for their significant clinical utility, as they are specifically designed to determine appropriate medication for a patient’s therapy on an individualized basis. There are commercially available and FDA-approved CDx tests based on plasma ctDNA NGS. For example, FoundationOne Liquid CDx allows qualitative measurement of the insertions, deletions, substitutions, rearrangements, and copy number changes in over 300 genes. This test is valuable for identifying effective therapies for several cancers, including PC; however, it does have limitations, such as the risk of false-negative results and inability to detect certain variations [130]. The cost of a single test is $3500 [155]; however, due to the insurance coverage received by 87% patients in the United States, the test comes at zero expense to most individuals [156].

Typical digital PCR-based platforms include ddPCR and BEAMing (beads, emulsions, amplification, and magnets), which are both recognized for their increased sensitivity and multiplexing capabilities [112]. A test based on the BEAMing method, which combines digital PCR and flow cytometry, has been utilized in a clinical trial of a CYP11A1 inhibitor—a new class of drugs that shows promise for mCRPC treatment [157].

In addition, the recent developments include a machine-learning tool designed to predict whether the ctDNA analysis would be more effective than tissue biopsy analysis based on the results of laboratory and radiographic tests [118].

### 4.4. Challenges and Future Directions

Several barriers exist to the widespread usage of ctDNA assays in clinical practice. To date, most of the studies have been correlative and exploratory, highlighting the need for prospective, biomarker-based studies to evaluate the clinical utility of these assays [158]. Recently published ASCO guidelines have recommended ctDNA testing for repeat assessments of patients whose previous results were negative or inconclusive, particularly when there has been a significant change in clinical status or when the metastatic site was unavailable for biopsy [115]. Overall, the information provided by ctDNA analysis was deemed not yet sufficiently practical to guide clinical decision-making.

The ctDNA levels in blood decrease with effective treatment and increase with disease progression, whether due to relapse, development of resistance, or initial treatment ineffectiveness. Notably, the correspondence of genomic variation profiles—specifically, CNVs—between tissues and ctDNA also varies with disease progression. This correspondence fluctuates around 50–60% in localized and low-metastatic cancers. In mHSPC, the correspondence is 80%, while in mCRPC, it can exceed 90% [109].

Low ctDNA concentrations increase the probability of false-negative results when analyzing individual alleles. Therefore, without developing more sensitive analytical methods, the reliability of such data remains insufficient for clinical use. At the same time, maintaining the specificity is challenging, as these two factors can conflict [159]. Additionally, the issue of low proportions of desired sequences exists at the level of individual gene variations, which can have frequencies below the detection threshold, but still possess clinical significance [123]. Genetic variations detected in ctDNA can originate not only from cancer tissues but also from other sources, such as hematopoietic cells. Identifying such sequences is impossible when comparing the ctDNA with the healthy part of the genome [160].

Another obstacle is the cost-effectiveness of the deep sequencing methods, with a limited ability to detect patient-specific mutations in the more affordable methods, such as ddPCR/BEAMing [161]. ASCO notes that NGS testing is available to a minority of patients due to its high cost. At the same time, the liquid biopsy-based tests do not have a significant difference in their availability compared to the tissue biopsies [115]. Potential solutions include developing alternative analysis methods, improving existing sequencing methods to lower costs, and accumulating sufficient data on significant biomarkers. This would optimize targeted methods for mass use in medical institutions.

Among recent studies, there has been significant heterogeneity in the protocols at all stages of analysis, including pre-analytical conditions, such as sample collection, storage, transportation, and processing [112,161]. For instance, a widely used approach for cfDNA isolation involves centrifugation to remove the blood cells, which prevents their lysis and the subsequent release of genomic DNA. This release can increase the total concentration of the cfDNA of wild-type sequences, thereby reducing the proportion of ctDNA [162]. The key factors for maintaining ctDNA levels above the LoD include minimizing the processing time after blood collection and collecting the blood in tubes containing EDTA to inactivate DNase. The DNA isolation and purification can be performed using various methods, including phenol–chloroform extraction, silica gel membrane columns, and magnetic beads [163]. Numerous commercial kits have been developed based on these methods, each featuring advantages and disadvantages. To enhance the reliability and reproducibility of the analysis, it is essential to standardize the analysis protocols and validate them according to the international standards [164].

## 5. Discussion

Circulating tumor DNA analysis has considerable potential for integration into clinical practice. However, its widespread clinical implementation is currently constrained by several significant limitations. The key challenge of the ctDNA analysis lies in the low abundance of ctDNA, particularly in patients with the early-stage or localized disease, where the tumor burden is limited and the fraction of tumor-derived DNA in circulation can be minute. This scarcity complicates the reliable detection and accurate quantification of ctDNA, necessitating the development and deployment of highly sensitive and specific analytical platforms. The current methodologies often require advanced technologies, such as targeted NGS, digital PCR, or novel methylation-based WGS assays, which can be costly and technically demanding.

The lack of standardized protocols for sample collection, processing, and data analysis hampers the reproducibility and comparability of the results across studies and clinical settings. Pre-analytical variables, including blood collection tubes, processing times, and DNA extraction methods, can significantly influence the ctDNA yield and integrity, further complicating the assay performance. Tumor heterogeneity, both spatial and temporal, can result in variable ctDNA shedding and diverse mutational or epigenetic profiles, making it difficult to capture a comprehensive molecular portrait of the disease from a single blood draw. Furthermore, the clonal hematopoiesis and other non-tumor-derived sources of cell-free DNA can confound interpretation of the ctDNA signals, potentially leading to false positives or misclassification.

Despite these hurdles, rapid technological advancements have been steadily improving the sensitivity, accuracy, and accessibility of ctDNA-based assays. The innovations in sequencing, combined with machine learning, enhance detection of low-frequency variants, fragmentation patterns, and epigenetic signatures. Concurrently, efforts to harmonize the protocols and validate the assays are underway, which will be critical for regulatory approval and routine clinical implementations. With continued research, robust clinical validation, and ongoing technological innovation, ctDNA analysis holds significant promise to transform the diagnostic and therapeutic landscape of prostate cancer. It offers the potential for more precise disease detection, real-time monitoring of treatment response, early identification of resistance mechanisms, and ultimately, delivery of more timely, accessible, and personalized patient care.

## 6. Conclusions

Prostate cancer remains a highly prevalent and clinically challenging malignancy primarily due to the limitations of current diagnostic tools. Liquid biopsy is a promising approach for more accurate, minimally invasive, and dynamic analysis. As one of the biomarkers for liquid biopsy, ctDNA reports on tumor-specific genetic and epigenetic features, which enables early diagnosis of PC, real-time monitoring of disease progression and treatment response, and even detection of resistance mutations. However, the clinical applications of ctDNA analysis require further optimization and adaptation. With continued research, ctDNA analysis holds significant promise for transforming the prostate cancer diagnostic and therapeutic landscape.

## Figures and Tables

**Table 1:** Summary of the Metastatic Patterns by Sites and Prognostic Significance.

Site	Prevalence [10]	Prognostic Significance [11,12]
**Bone**	90%	Associated with skeletal-related events (e.g., fractures)
**Lymph Nodes**	84%	Poor response to local therapies
**Lungs**	46%	Often concurrent with bone metastasis
**Liver**	25%	Halves median survival vs. bone-only
**Pleura/Adrenals**	21%/13%	Rarely isolated

**Table 2:** Main imaging techniques used for mPC detection.

Modality	Mechanism and Utility	Clinical Role	Limitations	Additional Notes
**Bone Scan** [23]	Uses radioactive tracers that accumulate in osteoblastic metastases, creating “hot spots”.	Gold standard for detecting osteoblastic bone metastases; cost-effective and widely available.	- Low sensitivity for early micrometastases or osteolytic lesions. - False positives (e.g., fractures).	Serial scans monitor treatment response, though changes lag behind PSA kinetics.
**CT Scan** [24]	Cross-sectional imaging to visualize lymphadenopathy, visceral metastases (liver, lungs), and soft tissue.	Part of TNM staging; added tool for diagnostics and treatment planning.	Limited sensitivity for subcentimeter lesions or early lymph node/bone metastases.	Often paired with MRI for comprehensive assessment.
**MRI** [24]	- Does not involve X-rays. - Superior in soft-tissue contrast. - MRI/US-fusion-targeted prostate biopsies.	- Defining the extent of local disease, differentiation of organ-confined disease. - Multiparametric MRI improves specificity.	Less sensitive than PSMA PET/CT for small bone/lymph node metastases.	Preferred for assessing soft-tissue recurrence in the prostate bed.
**PSMA PET/CT** [26,27,28]	Targets PSMA (overexpressed in prostate cancer) using tracers, such as gallium−68, fluorine−18, etc.	- Detects micrometastases (<10 mm) missed by conventional imaging. - Frequently alters management (up to 33% of cases).	- Limited availability and higher cost. - May miss PSMA-negative tumors (rare).	Recommended by NCCN/EUA guidelines for high-risk primary staging and biochemical recurrence.
**FDG PET/CT** [29]	Detects glucose metabolism via fluorodeoxyglucose (FDG) uptake in metabolically active tumors.	Limited role in prostate cancer due to low FDG avidity in most adenocarcinomas.	- Limited ability to detect micrometastases (<2 cm). - Low sensitivity in PC. - FDG accumulation in non-cancerous tissues.	Occasionally used in patients with advanced disease.
**PSMA PET/MRI** [25]	Combines PSMA-targeted PET with MRI’s soft-tissue resolution for hybrid metabolic/anatomic imaging.	- Enhances detection of small bone/lymph node metastases. - Improved precision in diagnostics, treatment planning.	Limited availability, longer scan time, and higher cost than PET/CT.	Emerges as a “one-stop” modality for high-risk patients, but lacks widespread adoption.
**Whole-Body MRI** [30]	Multi-sequence MRI covering entire body without radiation; detects bone/soft-tissue metastases.	- Alternative to bone scans for osteolytic/osteoblastic lesions. - Useful in radiation-free monitoring.	- Long scan time (30–60 min).	Preferred young patients or those requiring repeated imaging (e.g., active surveillance).

**Table 3:** Diagnostic Performance of Cell-Free DNA Biomarkers in Localized Prostate Cancer.

Biomarker	Sample Type	Sensitivity (95% CI)	Specificity (95% CI)	PPV (95% CI)	NPV (95% CI)	Purpose	Study
**Panel of 10 epigenetic biomarkers**	Post-digital rectal examination urine	81% (0.68–0.93)	76% (0.63–0.88)	71% (N/A)	85% (N/A)	Diagnosis	[75]
	First void urine	93% (0.84–1.02)	77% (0.63–0.91)	77% (N/A)	93% (N/A)	Diagnosis; risk stratification	
**Panel of six** **methylated markers**	Post-digital rectal examination urine	89% (0.79–0.97)	71% (0.57–0.86)	>70% (N/A)	≥90% (N/A)	Diagnosis; risk stratification	[76]
	First void urine	94% (0.84–1.0)	71% (0.57–0.86)	>70% (N/A)	≥90% (N/A)	Prognosis —metastasis risk	
**RASSF2**	Serum	69% (0.39–0.91)	39% (0.24–0.55)	26% (0.19–0.36)	80% (0.62–0.91)	Diagnosis	[77]
**cfDI** **(ALU 247/115 ratio)**	Plasma	81.7% (N/A)	78.8% (N/A)	89% (N/A)	67% (N/A)	Diagnosis	[80]
**cfDI**	Urine	79% (0.62–0.90)	84% (0.65–0.94)	N/A	N/A	Risk stratification	[79]
**cfDI** **(ALU 247/115 ratio)**	Serum	N/A	N/A	N/A	N/A	Diagnosis; risk stratification	[81]
**cfDI** **(ALU 247/115 ratio)**	Plasma	N/A	N/A	N/A	N/A	Diagnosis (not significant)	[82]
**cfDNA methylome**	Plasma	N/A	N/A	N/A	N/A	Diagnosis —aggressive PC	[74]
**RARβ2 promoter methylation**	Seminal fluid	N/A	N/A	N/A	N/A	Diagnosis —high-grade PC	[87]
**CAV1 (CpG1)**	Seminal fluid	59% (0.51–0.75)	63% (0.51–0.75)	N/A	N/A	Prognosis —biochemical recurrence	[88]
**LGALS3** **methylation**	Seminal fluid	56.4% (0.53–0.76)	70.4% (0.53–0.76)	N/A	N/A	Diagnosis	[89]

**Table 5:** Summary on ctDNA differences in localized vs. metastatic PC.

Parameter	Localized PC	Metastatic PC
**Fraction**	~0.1–10%, often below quantification threshold [108]	High (~50–95%), measurable for serial tracking [106]
**Markers in focus of research**	Epigenomic alterations and fragmentomics for early diagnosis and risk stratification [74,75,76,78,79]	ctDNAF, epigenomic, and genomic alterations, including alterations used in therapy selection—AR, HRR and MMR deficiencies, etc. [105,106,107,113]
**Methylation profiles**	Subtle, tissue-specific methylation signatures; harder to distinguish [74,75,76,77,87,88,89]	Abundant methylation aberrancies, patterns can differentiate between various subtypes [113]
**Tumor/ctDNA alterations correspondence**	~50–60% [109]	~80–90% [109]
**Assay requirements**	Ultra-sensitive [114]	Standard sequencing methods, low-pass sequencing, as well as ddPCR, etc. [106]
**Clinical utility**	Experimental; early detection [115]	Established for monitoring, diagnosis; being tested for therapy selection [115]

## Abbreviations

ADT	androgen deprivation therapy.
ALP	alkaline phosphatase.
ARSIs	androgen receptor signaling inhibitors.
AR	androgen receptor.
AR-V	androgen receptor variant.
BCR	biochemical recurrence.
BEAMing	beads, emulsions, amplification, and magnets.
BPH	benign prostatic hyperplasia.
BSP	bone sialoprotein.
CDx	companion diagnostics.
cfDI	cell-free DNA integrity index.
cfDNA	cell-free DNA.
cfMeDIP-seq	cell-free methylated DNA immunoprecipitation sequencing.
cfMBD-seq	cell-free methyl CpG-binding domain protein sequencing.
CgA	chromogranin A.
CIN	chromosomal instability.
CRPC	castration-resistant prostate cancer.
CRPC-NE	neuroendocrine castration-resistant prostate cancer.
CTCs	circulating tumor cells.
ctDNA	circulating tumor DNA.
ctDNAF	circulating tumor DNA fraction.
DDR	DNA damage repair.
ddPCR	digital droplet PCR.
dMMR	mismatch repair deficiency.
FDG	fluorodeoxyglucose.
GGT	gamma-glutamyl transferase.
Hb	hemoglobin.
HRR	homologous recombination repair.
HSPC	hormone-sensitive prostate cancer.
LoD	limit of detection.
mCRPC	metastatic castration-resistant prostate cancer.
mHSPC	metastatic hormone-sensitive prostate cancer.
mPC	metastatic prostate cancer.
MRD	minimal residual disease.
MSI	microsatellite instability.
NE	neuroendocrine.
NEPC	neuroendocrine prostate cancer.
NGS	next-generation sequencing.
NSE	neuron-specific enolase.
OPG	osteoprotegerin.
OPN	osteopontin.
OS	overall survival.
PAP	prostatic acid phosphatase.
PARP	poly(adenosine diphosphate-ribose)polymerase.
PARPis	poly(adenosine diphosphate-ribose)polymerase inhibitors.
PC	prostate cancer.
PFS	progression-free survival.
PSA	prostate-specific antigen.
PSMA	prostate-specific membrane antigen.
rPFS	radiographic progression-free survival.
RRM2	ribonucleoside-diphosphate reductase subunit M2.
RCA	rolling circle amplification.
SLFN11	schlafen family member 11.
SRE	skeletal-related event.
TMB	tumor mutation burden.
TP	transperineal.
TR	transrectal.
TRACP	tartrate-resistant acid phosphatase.
TRUS	transrectal ultrasound.
WGS	whole-genome sequencing.
